# Application Analysis of Combining BP Neural Network and Logistic Regression in Human Resource Management System

**DOI:** 10.1155/2022/7425815

**Published:** 2022-03-11

**Authors:** Ting Xiang, Ping Zhen Wu, Shihai Yuan

**Affiliations:** ^1^School of Management, Guangzhou Huashang College, Guangzhou 511300, China; ^2^Chinese Graduate School, Panyapiwat Institute of Management, Pak Kret, Thailand

## Abstract

Human resource management involves a variety of data processing, and the process is complicated. In order to improve the effect of human resource management, this paper combines BP neural network and logistic regression analysis to construct an intelligent human resource management system and uses backpropagation learning to adjust training errors and determine connection weights. Moreover, this paper estimates the probability of a certain event through regression analysis, predicts and analyzes the human resource management process, and builds an intelligent human resource management system with the support of joint algorithms. In order to explore the reliability of the joint algorithm proposed in this paper, the effectiveness of the algorithm proposed in this paper is verified through simulation tests. The experimental research results show that the human resource management system based on BP neural network and logistic regression proposed in this paper has good practical effects.

## 1. Introduction

With the development of the market economy, the competition between enterprises has also shown an increasingly fierce situation, and this kind of competition between enterprises is largely a competition for outstanding talents and human resources. For this reason, great attention should be paid to the management of human resources. At the same time, in order to achieve sustainable, healthy, and rapid development of enterprises, it is ultimately necessary to return to improving the level of human resource management, thereby enhancing the core competitiveness of modern enterprises themselves. With the integration of the world and economic globalization, in order to gain a place in the competition of modern society, companies not only need to reserve a large number of composite talents of various types of technology, management, and operation, but more importantly need to make full use of these human resources and effectively manage and rationally use these human resources to maximize their benefits [1]. Therefore, the management and development of human resources has very important practical significance in modern enterprises. The concept of human resource management first originated in Western countries and then spread to China. However, since it has not been a long time to enter the country, domestic enterprises are still in a stage of promotion and application in terms of human resources, and their maturity and perfection in this regard need to be improved. At the same time, although domestic experts started relatively late in human resource research compared with Western experts, Chinese scholars have also achieved certain results in human resource management research in recent years [2]. However, due to the far-reaching impact of traditional concepts, domestic companies still have some shortcomings in management methods. These concepts will inevitably hinder the further promotion and popularization of advanced human resource management technologies and methods in domestic companies. It is easy for domestic enterprises to stay at the initial level that needs to be improved in the use of human resource methods and the concept of human resource management. Therefore, from the perspective of most Chinese enterprises, especially some state-owned large-scale engineering construction enterprises, it is necessary to carry out the standardized construction of human resource management system [3].

Under the background of economic globalization, human resources, as a knowledge carrier, have been valued by organizations in the face of challenges such as highly developed technological changes and diversified customer needs. Under the influence of the “people-oriented” concept, in order to better promote the sustainable development of the organization, more and more organizations attach importance to human resource management and continuously improve their human resource competitiveness. However, the uniqueness of the organization and the complexity of people also make human resource management difficult. The innovation of the global economy and the development of society are constantly changing people's knowledge concept. People's concept of knowledge management has transitioned from the initial focus on its functions and characteristics to the application level of knowledge management. Human resource management is an objective guarantee for the development of a company. To achieve effective knowledge management, it must rely on the company's human resource management level and improve the company's human resource management development and operating capabilities. Applying knowledge management concepts to the company's human resource management is conducive to enhancing the company's human resource management and innovation capabilities.

This article combines BP neural network and logistic regression analysis to build an intelligent human resource management system and applies intelligent management methods to human resource management to improve the efficiency of human resource management.

## 2. Related Work

In order to make the research results of human resource management more practical and maneuverable, at present, the majority of foreign scholars' research on human capital has shifted from focusing on the macro level to more on the micro level. Starting from the micro level of the enterprise, these scholars mainly studied the relationship between enterprise human capital and enterprise performance.

The literature [4] believed that human resource management is affected by two aspects. One is the internal environment, including senior management's daily standards and values, corporate strategy, corporate culture, technology, structure, and scale. The other is the external environment, including the g economy/market, human diversity, values, laws, and competitors.

The literature [5] stated that the practice of human resource management is the product of a combination of multiple factors. The literature [6] believed that the influencing factors of human resource management in financial sectors such as banks include not only the market environment in which bank branches are located and the characteristics of bank employees, but also the recognition of employee contributions, performance evaluation and feedback systems, communication and exchanges between managers and employees, and other factors that have an important impact on the performance of banking institutions. The literature [7] found that there is a very important relationship between the implementation of employee training plans and performance. The literature [8] used years of education to represent general human capital, and years of work to represent enterprise-specific human capital. The results of empirical research found that there is a significant positive correlation between years of education and working years and the performance of different groups, and groups with higher education and rich experience of human capital have higher work performance. Literature [9] proposes an implementation model of integrated human resources management. This model summarizes and analyzes the functions of human resources management and ensures that the management of human resources can conform to the development strategy planning of the banking institution. The integrated management implementation model includes 9 interconnected steps of definition work, testing and appraisal, prediction and selection, orientation and training, and so on. At the same time, it is pointed out that if these nine steps are completed well, all three factors must be considered. The three factors are to have a banking culture system that aligns employees' personal goals with organizational goals, engages employees in the bank's senior management, and repositions the status of human resources experts.

In addition to the implementation model of integrated human resource management, literature [10] has conducted a lot of research on how banks conduct input resource management and believes that banks should improve input resource management procedures for human resource management, strengthen institutionalization, and establish effective integration The evaluation system summarizes the work status of each employee. At the same time, other departments of the bank should also cooperate with the human resource management plan to ensure the smooth progress of human resource management. Literature [11] studies the components of intellectual capital in the service industry and nonservice industry, namely, the relationship between human capital, structural capital, and customer capital, as well as their relationship with corporate performance. The research concluded that human capital has a positive effect on customer capital. The relationship between human capital and structural capital has different performances in different industries. Customer capital and structural capital have a significant positive correlation in both industries. Structural capital has a positive effect on corporate performance. Literature [12] replaces economic development with per capita GDP and relative growth rate and uses the enrollment rate of elementary, middle school, and higher education as a substitute variable for human capital. From the perspective of policy makers, it uses education consumption to affect the public. The ratio of total consumption is used as a substitute variable for human capital development. Literature [13] found that there is a significant causal relationship between people receiving elementary and middle school education and GDP per capita, while there is an opposite causal relationship between higher education and GDP per capita; that is, the development of higher education does not promote the growth of GDP. But the development of higher education is mainly driven by GDP growth. Literature [14] analyzes the effectiveness of human resource training and development and the effectiveness of the work of the human resource department through a large amount of data. it used single-factor variance to demonstrate his views through actual data, finally came to the importance of input resource development and investment, and proposed the importance of banks and other financial institutions for human resource investment in view of the particularity of human resource management in the banking sector significance. Literature [15] focuses on the input and output effects of human resources. Through research, it discovered the necessity of investing in human resources and believes that reasonable investment in human resources can ensure the realization of a company's long-term business strategic goals, especially the investment in technical talents that determines the prospects of an enterprise's development. Literature [16] pointed out that, generally speaking, the human capital status of a bank can be predicted to the development prospects of the bank organization, and a large amount of data and cases can be used to verify the important role of human resource management flexibility in the development of the organization. Literature [17] states that entrepreneurs' transnational experience is a kind of valuable and difficult to imitate human capital. Based on the collection of 256 multinational CEOs' transnational experience, they use empirical methods to prove the CEO's transnational experience. Special human capital has a positive and positive effect on company performance.

## 3. Human Resource Data Mining Algorithm Combining BP Neural Network and Logistic Regression

Data mining needs to be divided into several steps. Although different data research methods require different steps, they can still be summarized as the following general points: problem definition, data extraction, data preprocessing, knowledge extraction, evaluation, and interpretation of results.

Although data mining technology is a process of knowledge discovery, which aims to discover hidden information in redundant data, it is still possible to anticipate the results of the mining before data mining. This expectation is vague and not always achievable. Anticipating before data mining can guide the data mining process. The definition of the problem needs to be based on a full understanding of the relevant field and familiarity with the background knowledge. In this way, an effective mining target can be established [18].

Data mining is based on the information set, which is composed of data information. The acquisition of this information set requires a complex extraction process. Usually, through data query and compilation, the required data can be formed into a sample set. In addition, it is very important to use samples of which variables are included for data mining. An entity contains multiple attributes, and different attributes represent different entity characteristics from different sides. If the included attributes are consistent with the mining target, the probability of success is very high. If the included attributes are completely irrelevant to the mining target, no results can be obtained. However, if the sample includes enough attributes, useless attributes can be automatically excluded in the experiment, rather than being excluded in the inference process.

The preprocessing of data is a process of processing the extracted data, then checking whether the data is complete and consistent with the target, then processing the data noise, and deleting or filling the missing data.

The preprocessing of data must first solve the problem of noise and lack of data. The noise of the data can be summarized as duplication and numerical error. It is easy to solve the repeated data, but the wrong data depends on the experience and judgment of the analyst. Moreover, many of the original data are wrong in the lack of data. There are three solutions to the problem of missing data. The first is to discard records with missing values. The second is to replace missing values with median values for numerical missing values. The third is to replace missing values with data from other similar samples. This article adopts the first approach.

Through the use of selected data mining algorithms, the required knowledge is extracted from the data, which can be expressed in a specific or commonly used way. In the knowledge extraction stage, the processed data needs to be handed over to computer software for processing. According to different purposes, different data mining algorithms are selected for data mining, and finally the results of data mining are obtained.

Data mining technology emphasizes that the generalized knowledge can always be explained, but this explanation may not be available immediately. If we are always unable to find an explanation, then we should doubt the authenticity of knowledge. Therefore, we can think of data mining as a data analysis process of “determining goals-induction-interpretation.”

The results of data mining are not necessarily valuable. The data generated by activities under prescribed laws is not the result of random events, and the mining of these data is not necessarily valuable. In addition, if the results of data mining cannot be explained, it does not necessarily mean that a new kind of knowledge is obtained. It is likely that such mining is worthless. Therefore, data mining must not only follow the characteristics of the data mining technology itself, but also combine the characteristics of the data itself.

Artificial neural network is a parallel decentralized processing model that simulates the neural operation of the human brain. It is a nonlinear, adaptive information processing system composed of a large number of processing units. It has fault tolerance and learning ability, can deal with data omissions or errors, and can conduct self-learning and training to meet the needs of constantly changing actual conditions.

Neuron simplifies and simulates biological neuron and is the basic unit of neural network. The characteristics of a neural network are determined by the characteristics of neurons as its constituent parts. At present, there are many neuron models that have been proposed and are in use, and the most influential one is the M-P model, which was proposed by the psychologist MeCulloh and the mathematician Pitt. After continuous improvement by later scholars, a neural network model that is widely used in research and practice today has been formed. The information processing mechanism of neurons has the following six hypotheses [19]:

1. Each neuron is an information processing unit with multiple input and single output
2. There are two types of synapses: excitatory and inhibitory
3. Neurons have threshold characteristics and spatial integration characteristics
4. Time integration and refractory periods are ignored
5. The input and output processes of neurons have a fixed time lag, which depends on synaptic delay
6. Neurons are time-invariant; that is, synaptic strength and synaptic delay are constant

**Figure 1:**
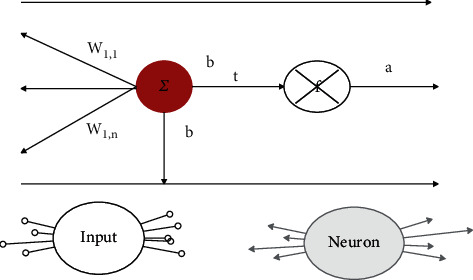
Neuron model with *R*-dimensional input.

A typical neuron model consists of five parts: input, network weights, summation unit, transfer function, and output. A typical R-dimensional neural network model is shown in Figure 1.

*P*_*i*_ represents the input of the original data, *W* is the network threshold, which is used to represent the correlation strength between the input and the neuron, and *b* is the neuron threshold with a general constant value of 1. As the summation unit, *n* is the first process of neuron processing input information. The weighted summation of the input signal is completed through *n*, that is, *n* = *w* ^∗^ *p* + *b*. The second process in which the neuron processes the input signal is to use the transfer operation unit *f* to perform function calculation on the summation unit to obtain the output of the neuron. After using the input and output vectors and the weight vector to perform weighted summation and function action, the final output is obtained, which can be expressed by the following function: *a* − *f*(*w* ^∗^ *p* + *b*).

**Figure 2:**
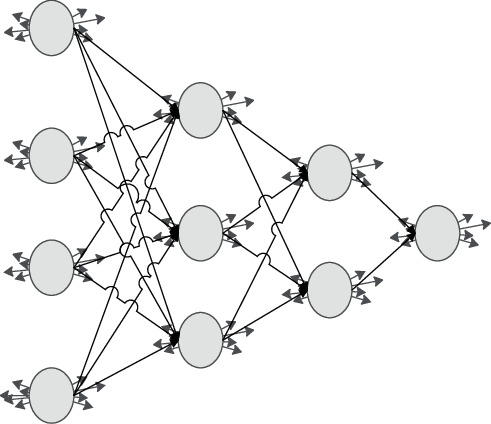
Neuron network structure with four input nodes and two hidden layers.

In this article, the forward three-layer BP neural network model is used. As you can see above, a complete neural network is composed of an input layer, a hidden layer, and an output layer. Each node of the input layer represents a predictor variable, the output layer represents multiple target variables, and the hidden layer is located between the input layer and the output layer. The training of the neural network determines the specific structure of the hidden layer, but for users of this network model, the training process and the specific structure of the hidden layer are not known. There are two determinants of the complexity of this model: one is the number of nodes in the hidden layer, and the other is the number of hidden layers.  Figure 2 depicts a neural network with four input nodes and two hidden layers [20].

Feedforward neural network refers to a neuron network in which each neuron node of each layer transmits its results to each neuron node of the next layer. The arrows in the above figure represent data transfer. While these arrows indicate the transfer relationship, they also each have a coefficient. This coefficient indicates the degree of influence of the output of the previous node on the next node.

The feedforward neural network model is established through continuous training. By continuously inputting each sample into the model, the coefficients are adjusted according to the error between the calculation result and the observation result, until the accuracy of the model reaches an acceptable level. This adjustment is carried out through algorithms. Among various learning algorithms, the Error Backpropagation Algorithm is widely used.

The BP algorithm was first proposed by [21, 22] developed the theory. The BP algorithm is a guided learning process. In the training phase, in order to minimize the training error rate, the training samples repeatedly pass through the network while modifying the link weight to continue the network training until a specific condition is reached. This condition can be the minimum error that the network converges to, the maximum number of repetitions, or a specific time standard. The BP algorithm includes the following four steps [23]:

Mode forward propagation: the algorithm passes from the input layer to the output through the hidden layer.
Error backpropagation: the algorithm transmits the error signal of the difference between the expected output and the actual output from the output layer through the hidden layer to correct the connection weight.
Memory training: mode forward propagation and error backward propagation are repeated.
Learning convergence: the algorithm makes the overall error of the network tend to a minimum after repeated learning.
The most important process in the BP algorithm is to adjust the training error and determine the connection weight through backpropagation learning. The processing logic of the backpropagation mode is as follows.
Initialization:
  (1) The algorithm randomly determines a coefficient of [-1,1] for every two adjacent neurons.
  (2) The algorithm specifies a learning parameter *r* of [0,1].
  (3) The algorithm determines a learning termination condition, which is usually expressed as
    (1) $$\begin{array}{c} ms=\frac{\sum_{n}\sum_{i}ABS\left(T_{ni}-O_{ni}\right)}{ni}<0.1, \end{array}$$
  *T* is the observation value, O is the output value, *n* is the number of samples, and *i* is the number of input attributes.
Training data:
  (1) Each neuron does the following processing on the received data:
    (2) $$\begin{array}{c} Y=\sum_{j=1}^{m}q_{j}\times O_{j},\\ O=\frac{1}{1+e^{-y}}. \end{array}$$
  Among them, *q*_*i*_ is the coefficient between the neuron that transmits the data to the current neuron, and *O*_*j*_ is the data transmitted by the previous node. In order to continue the transmission, the algorithm uses the second formula to convert *Y* into [0, 1].
  (2) The algorithm determines the difference caused by the calculation result ERROR()
    (3) $$\begin{array}{c} ERROR\left(k\right)=\left(T-O_{k}\right)\times O_{k}\times \left(1-O_{k}\right). \end{array}$$
  (3) The algorithm adjusts the coefficient:
    (4) $$\begin{array}{c} \Delta q_{jk}=r\times ERROR\left(k\right)\times O_{j},\\ q_{jk}\left(new\right)=q_{jk}\left(current\right)+\Delta q_{jk},\\ ERROR\left(j\right)=\left(\sum_{k}ERROR\left(k\right)\times q_{jk}\right)\times O_{j}\times \left(1-O_{j}\right). \end{array}$$
  If the termination conditions are not met, the above steps are always repeated.

Logistic regression model is a nonlinear regression model in which the response function is S or inverted S, and the probability value of the response function is between 0 and 1. The occurrence of a random event is often related to multiple influencing factors. Logistic regression analysis can be used to estimate the probability of a certain event. Before using the model to make predictions, it is necessary to filter out the factors that have an impact on the probability of the event from many factors that may affect the event and use these factors to model.

The logistic regression model is as follows:

(5) $$\begin{array}{c} Y_{1}=\ell_{1}+X_{11}\times \beta_{1}+X_{12}\times \beta_{2}+X_{13}\times \beta_{3}+......+X_{1i}\times \beta_{i},\\ Y_{2}=\ell_{1}+X_{21}\times \beta_{1}+X_{22}\times \beta_{2}+X_{23}\times \beta_{3}+......+X_{2i}\times \beta_{i},\\ Y_{3}=\ell_{1}+X_{31}\times \beta_{1}+X_{32}\times \beta_{2}+X_{33}\times \beta_{3}+......+X_{3i}\times \beta_{i},\\ Y_{n}=\ell_{1}+X_{n1}\times \beta_{1}+X_{n2}\times \beta_{2}+X_{n3}\times \beta_{3}+......+X_{ni}\times \beta_{i}. \end{array}$$

Among them, *Y* is a vector of *n* × 1, and the element *Y*_*i*_ is a dichotomous variable. *X* is the matrix of nxi, and *X*_*ni*_ is the value of the *n*-th sample and the *i*-th explanatory variable. *β* is the vector of *i* × 1, which is the coefficient of the explanatory variable.

The logistic model assumes that the probability of *Y* taking a certain value is *T*.

(6) $$\begin{array}{c} p_{i}=\frac{\exp\left(X_{i}\beta \right)}{1+\exp\left(X_{i}\beta \right)}. \end{array}$$

In the analysis of this article, *Y* = 0 represents a financial crisis company, *Y* = 1 represents a normal company, *X* is a risk measurement index, and *β* is a regression coefficient, *Р* represents the probability that a company with *Y* = 0 may have a financial crisis, and *P*_1_=1 − *P* represents the probability that a company with *Y* = 1 will not have a financial crisis.

(7) $$\begin{array}{c} p_{1}\left(Y_{i}=1\right)=\frac{\exp\left(\alpha +\beta_{1}x_{1}+\beta_{2}x_{2}+......+\beta_{k}x_{k}\right)}{1+\exp\left(\alpha +\beta_{1}x_{1}+\beta_{2}x_{2}+......+\beta_{k}x_{k}\right)},\\ p_{0}\left(Y_{i}=0\right)=1-p_{1-}\frac{1}{1+\exp\left(\alpha +\beta_{1}x_{1}+\beta_{2}x_{2}+......+\beta_{k}x_{k}\right)}. \end{array}$$

We choose 0.5 as the cut-off point, *P* > 0.5 corresponds to ST companies, and *P* < 0.5 corresponds to non-ST companies.

## 4. Human Resource Management System

**Figure 3:**
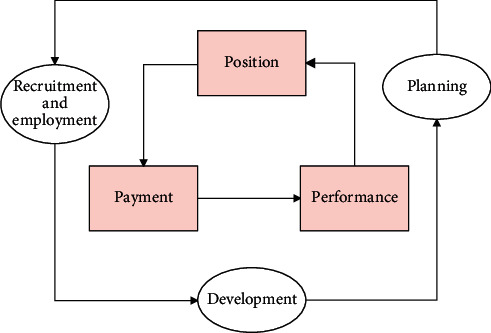
Human resource management based on 3*P*.

For the purpose of reducing management costs, it is impossible to conduct comprehensive and standardized human resource management. Therefore, only by grasping the key to human resource management and fully embodying the core and essence of modern human resource management “understanding human nature, respecting human nature, and people-oriented” in terms of job responsibilities, job evaluation, and salary distribution, can we get out of the dilemma of human resource management and embark on a more standardized track. Therefore, the 3*P* management model is more suitable for the practical operation, and the design of our core modules is also based on 3*P*, as shown in Figure 3.

**Figure 4:**
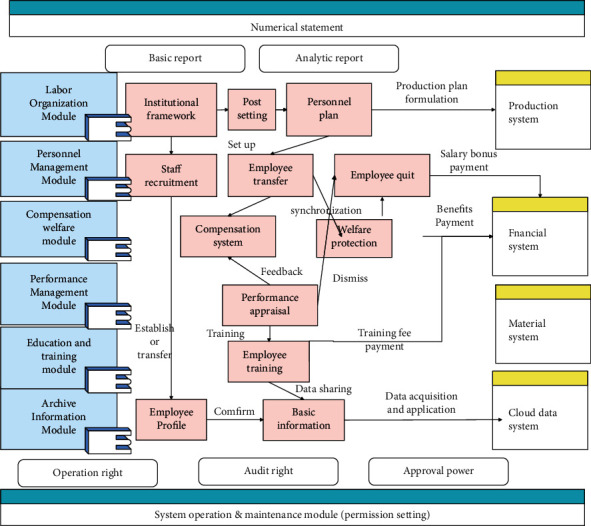
System frame diagram.

The basic architecture diagram of the system is drawn up, as shown in Figure 4.

**Figure 5:**
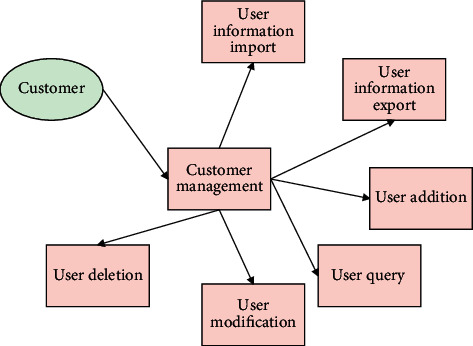
Use case diagram of system function management.

User management can be divided into two categories: system special users (system administrators) and nonadministrator users. The system administrator can manage all users in the system, including modifying user information, querying user information, adding user information, and deleting user information. The system administrator use case diagram is shown in Figure 5.

**Figure 6:**
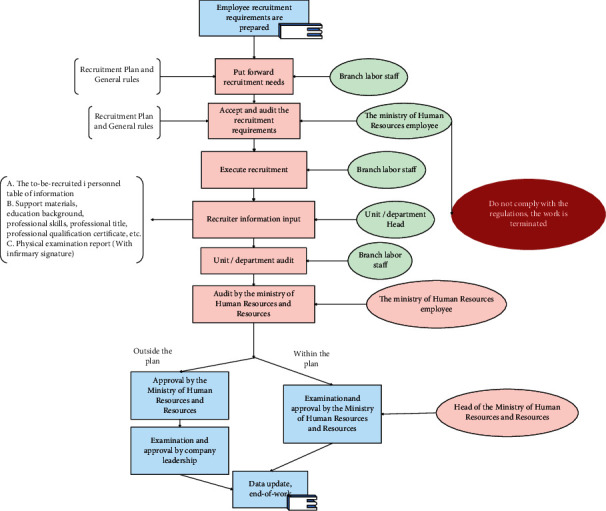
Logic diagram of employee recruitment operation.

**Figure 7:**
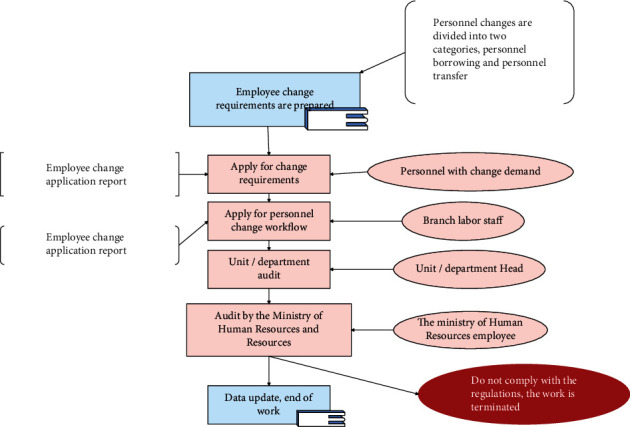
Logic diagram of staff transfer operation.

**Figure 8:**
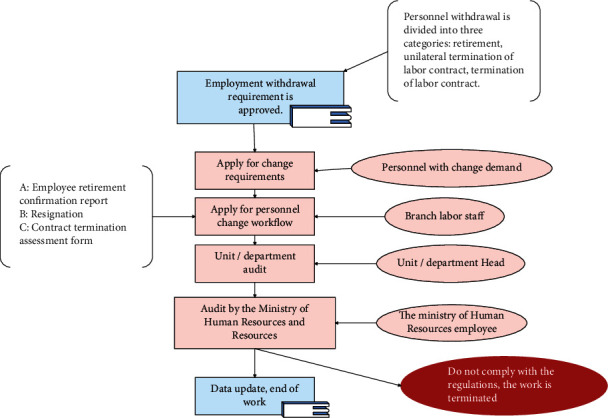
Logic diagram of employee exit operation.

Figures 6–8 are operational logic diagrams reflecting the relevant personnel flow in the HRMS system. The source is the workflow chart of the actual management of the human resources department, which is the true reaction of the work process, and reflects the unity of the human resource management HRMS system and the actual business.

The human resource management system proposed in this paper uses BP neural network and Logistic regression joint algorithm to manage human resources. In order to explore the reliability of the joint algorithm proposed in this paper, the effectiveness of the algorithm in this paper is verified through simulation tests. The experiment uses the same human resource management system, and only the data mining algorithm is different. The experiment set up three sets of variables, respectively, namely, the joint algorithm, the BP neural network algorithm, and the logistic regression algorithm, to verify the effects of these three methods on human resource management, and the results are shown in Table 1.

From the above research, it can be seen that the joint algorithm proposed in this paper is significantly higher than the single algorithm in human resource management data mining. On this basis, this paper studies the human resource management effect of the human resource management system based on the joint algorithm and conducts the research through expert evaluation. The results obtained are shown in Table 2.

**Figure 9:**
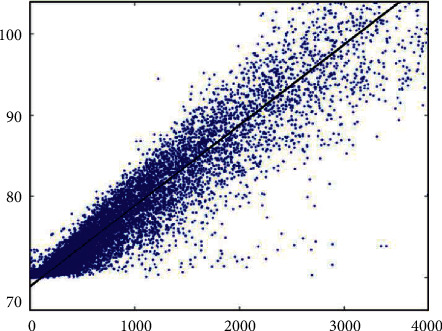
Regression analysis results.

Through massive data, regression analysis with the data in this article, the results shown in Figure 9 are obtained.

**Figure 10:**
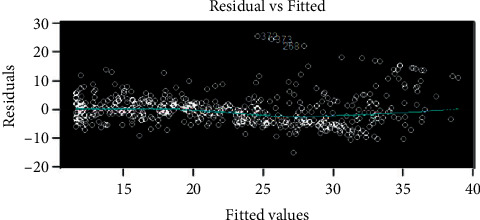
Model residual analysis results.

Perform residual analysis on the model and get the result as shown in Figure 10.

From the above research, we can see that the human resource management system based on BP neural network and logistic regression has good practical effects.

## 5. Conclusion

In order to gain a competitive advantage in human resource management, an organization needs to understand its connotation deeply and find a management form and mode that matches the organizational structure. It is necessary to combine scientific methods and certain material resources, use scientific and reasonable methods to train and deploy manpower, and induce their behaviors, thoughts, and behaviors appropriately. Moreover, it is necessary to mobilize people's subjective initiative to maintain the optimal ratio of human and material resources, so as to achieve the process of organizational goals, that is, human resource management. Human resource management is conducive to giving full play to the advantages of the organization's talents and realizing that people make the best use of their talents and get what they want. Data mining management can improve the quality of the company's employees, thereby enhancing the company's comprehensive competitiveness. At the same time, it helps eliminate some of the current human resource management problems in some organizations, such as low professionalism, chaotic recruitment procedures, imperfect incentive mechanisms, and training systems, and can improve the company's human resource management level and ability. This article combines BP neural network and logistic regression analysis to build an intelligent human resource management system and applies intelligent management methods to human resource management to improve the efficiency of human resource management.

## Figures and Tables

**Table 1:** Data mining effect test.

Number	BP neural network	Logistic algorithm	Joint algorithm
1	66.94	78.93	88.48
2	70.48	65.34	84.61
3	73.94	69.03	89.06
4	69.39	74.49	86.56
5	74.84	77.39	88.11
6	61.25	63.62	85.41
7	75.15	67.03	91.83
8	67.43	71.42	84.83
9	67.43	65.10	88.81
10	71.18	68.68	86.06
11	74.90	80.27	87.47
12	69.35	73.64	83.74
13	68.54	68.81	92.88
14	67.62	75.26	84.71
15	77.74	65.08	83.39
16	68.73	67.13	85.50
17	73.47	68.53	83.46
18	75.57	80.51	90.43
19	67.60	75.48	83.62
20	63.86	64.60	89.28

**Table 2:** The results obtained.

Number	Management evaluation	Number	Management evaluation	Number	Management evaluation
1	89.38	21	91.01	41	88.07
2	87.26	22	94.20	42	94.27
3	86.62	23	87.05	43	92.28
4	92.90	24	90.85	44	88.31
5	87.37	25	87.44	45	86.81
6	93.75	26	86.35	46	87.91
7	88.73	27	87.92	47	93.98
8	88.38	28	89.40	48	94.32
9	91.05	29	88.20	49	93.61
10	92.31	30	87.35	50	88.08
11	87.25	31	94.33	51	94.06
12	90.94	32	94.18	52	89.51
13	94.98	33	87.05	53	90.66
14	92.85	34	86.51	54	91.26
15	93.76	35	93.66	55	88.08
16	93.30	36	87.47	56	88.88
17	89.95	37	87.73	57	91.62
18	90.58	38	94.50	58	93.11
19	93.01	39	86.15	59	91.85
20	87.77	40	90.28	60	92.40

## Data Availability

The data used to support the findings of this study are included within the article.
